# Oral microbiota in human systematic diseases

**DOI:** 10.1038/s41368-022-00163-7

**Published:** 2022-03-02

**Authors:** Xian Peng, Lei Cheng, Yong You, Chengwei Tang, Biao Ren, Yuqing Li, Xin Xu, Xuedong Zhou

**Affiliations:** 1 State Key Laboratory of Oral Diseases, West China Hospital of Stomatology, National Clinical Research Center for Oral Diseases, Sichuan University, Chengdu, China; 2 Department of Cariology and Endodontics, West China Hospital of Stomatology, Sichuan University, Chengdu, China; 3 Department of Obstetrics and Gynecology, West China Second University Hospital, Sichuan University, Chengdu, China; 4 Department of Gastroenterology, West China Hospital, Sichuan University, Chengdu, China

**Keywords:** Microbiology, Diseases

## Abstract

Oral bacteria directly affect the disease status of dental caries and periodontal diseases. The dynamic oral microbiota cooperates with the host to reflect the information and status of immunity and metabolism through two-way communication along the oral cavity and the systemic organs. The oral cavity is one of the most important interaction windows between the human body and the environment. The microenvironment at different sites in the oral cavity has different microbial compositions and is regulated by complex signaling, hosts, and external environmental factors. These processes may affect or reflect human health because certain health states seem to be related to the composition of oral bacteria, and the destruction of the microbial community is related to systemic diseases. In this review, we discussed emerging and exciting evidence of complex and important connections between the oral microbes and multiple human systemic diseases, and the possible contribution of the oral microorganisms to systemic diseases. This review aims to enhance the interest to oral microbes on the whole human body, and also improve clinician’s understanding of the role of oral microbes in systemic diseases. Microbial research in dentistry potentially enhances our knowledge of the pathogenic mechanisms of oral diseases, and at the same time, continuous advances in this frontier field may lead to a tangible impact on human health.

## Introduction

**Fig. 1:**
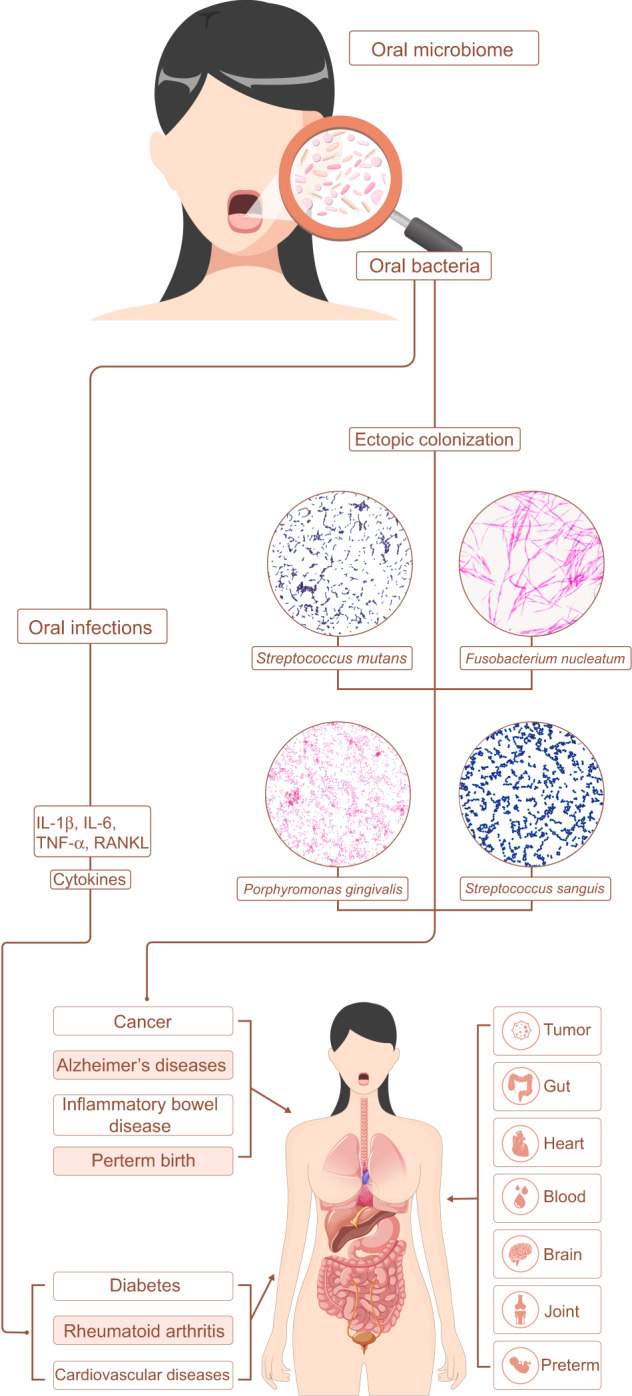
Schematic of the oral microbiota in human systematic diseases. Oral microbes affect the process of systemic diseases through the inflammatory response caused by oral infection or the ectopic colonization of oral microorganisms in other organs or tissues of the human body, such as tumor, gut, heart, blood, brain, joint, placenta.

In 1891, the first oral microbiologist Willoughby D. Miller put forward the theory of oral focal infections, suggesting that oral microbial infection can affect other parts of the body, related to a variety of systemic diseases.^1^ Frank Billings speculated that the infection of teeth may be the cause of rheumatoid arthritis, nephritis, endocarditis, and other diseases.^2^ Proponents of this theory believe that dental plaque and its metabolites can enter the blood circulatory system and cause a variety of systemic or degenerative changes. Therefore, the treatment of systemic diseases by extracting the affected tooth is not only popular in dentistry, but also entire medical field. However, the theory of oral focal infection has not received enough attention and theoretical support. With the advances of microbiome research, the association between oral microbes and a variety of human chronic diseases has been studied, including inflammatory bowel disease,^3^ cancers,^4^ cardiovascular diseases,^5^ Alzheimer’s disease,^6^ diabetes,^7^ rheumatoid arthritis,^8^ and preterm birth^9^ (Fig. 1). In addition, the changes of oral microbiota in the state of systemic diseases are gradual and repeatable. Therefore, oral microbes can reflect human health and disease status in real-time and have important value in disease risk early warning and curative effect prediction.

Over 700 kinds of microorganisms are colonized in the human oral cavity.^10^ The oral microbiome is one of the most important and complex microbial communities in the human body and is also one of the five research priorities (oral cavity, nasal cavity, vagina, intestine, skin) of the human microbiome project (HMP).^11^ With the consummation of the human microbiome project, the understanding of oral microbes has become more in-depth, and it is not limited to further understanding the role of oral microorganisms in caries, periodontal diseases, and other oral diseases. Evidence is increasingly inclined to believe in the oral lesion theory proposed by Miller. The inflammation of periodontitis leads to the loss of connective tissues and bones.^12^ Extensive inflammatory cell infiltration appears in the connective tissue near the periodontal pocket epithelium.^13^ It is generally believed that this low-grade inflammation will disturb the health of the whole body or worsen other systemic diseases.^14^ Therefore, in the general population, chronic periodontitis may be an important source of invisible peripheral inflammation. Thus, periodontitis is also called “low-grade systemic disease”, affecting a variety of systemic diseases.

Particularly, a large amount of evidence has proved that bacteria are closely related to tumor development in the past two decades.^15^ For example, the role of human papillomavirus in oral cancer,^16^ *Helicobacter pylori* in gastric cancer,^17^ *Chlamydia pneumoniae* in lung cancer,^18^ *Salmonella typhi* in gallbladder cancer,^19^ *Streptococcus bovis*,^20^ *Bacteroides fragilis*^21^, and especially the periodontal pathogen *Fusobacterium nucleatum* in colon cancer.^22^ These studies have led to the possible role of bacteria in the occurrence of tumors, and the subsequent research results do provide some evidence to support it. There is a lot of evidence that oral microorganisms can induce cancer through direct or indirect factors.^23^ For example, oral microorganisms can secrete polysaccharides or use their flagella to accumulate on the surface of tumor cells in large numbers, induce chronic inflammation, and the secretion of cytokines directly promotes the growth of tumor cells.

Increasing evidence supports the association between the oral microbiome and human systemic diseases.^24^ This association may be attributed to the ability of many oral microbes to influence the inflammatory microenvironment. Excluding unfavorable factors such as physical activity, poor oral condition is closely related to unhealthy body index. Clinical and basic research on oral health and systemic diseases has become a frontier hotspot. Herein, we reviewed advances in the relationship between oral microbes and digestive diseases, cancers, cardiovascular diseases, Alzheimer’s disease, diabetes, rheumatoid arthritis, and preterm birth (Fig. 1).

## Oral microbes and digestive system diseases

The possible pathways for oral microbes to act on the intestinal tract include that the oral microbes invade the intestines, causing imbalances in the intestinal microecology and affecting organs of the digestive system.^25^ For example, the colonization of oral microbes affects the metabolism of butyrate of intestinal microbes^26^; oral microbes, especially periodontitis pathogens, can enter the bloodstream through periodontal inflammation tissues and enter the systemic circulation, thereby acting on the whole body.^27^ As mentioned in the study of colorectal cancer, *F. nucleatum* colonizes the intestine and acts through the blood pathway.^28^ In addition, the metabolites of oral microorganisms enter the systemic circulation through the blood, which makes a low-grade inflammation in the human body, and promotes the occurrence and development of chronic inflammatory diseases in the digestive system.^29^ This approach is gradually supported by the evidence of oral microbiome research, and it is widely recognized in the research of systemic diseases caused by the imbalance of the intestinal flora. Therefore, this approach may also be the main role of oral microbes in the digestive tract and an important way to affect systemic diseases.

### Oral microbes and inflammatory bowel disease

Adults produce more than 1000 mL of saliva every day, almost all of which enter the gastrointestinal tract.^30^ Therefore, oral microbes, as an important reservoir of intestinal microbes, play an important role in maintaining the internal stability of the intestinal microecosystem. The virulent strains in the oral cavity migrate to the intestine through the digestive tract or blood, which affects the process of many intestinal inflammatory diseases.^31^ Inflammatory bowel disease (IBD) is a global disease, especially in developed countries, the prevalence in developing countries is also increasing year by year, the prevalence in China is about 3.44 per 100,000 persons.^32^ Chronic non-specific intestinal inflammatory diseases, whose etiology is not well understood, are believed to be determined by genetic and environmental factors.^33^ The intestinal microbiome also plays an important role during IBD.^34^ At the same time, recent studies found a correlation between oral microbes and IBD.^3^

Periodontal disease is considered to be a risk factor for a variety of systemic diseases.^35^ *Porphyromonas gingivalis* and *F. nucleatum* are the main pathogens of periodontal disease.^36^ The inflammation caused by *P. gingivalis* in the oral cavity can lead to the disorder of the intestinal microbial community structure, the destruction of the intestinal barrier, the induction of endotoxemia, and the systemic inflammatory response.^37^ Under normal circumstances, *F. nucleatum* is almost impossible to detect in the intestine, but the bacteria can migrate to the intestine, inhibit the immune response mediated by T cells, thus promoting the progress of IBD.^38^ In addition, *Streptococcus salivarius* is an early colonizer in the oral cavity, which also can colonize the intestinal tract, down-regulate the nuclear transcription factor NF-кB of small intestinal epithelial cells and participate in the process of intestinal inflammation and homeostasis.^39^ Through population surveys, it is found that poor oral care behaviors related to alteration of the oral flora, cause an imbalance of intestinal microbes, and lead to the occurrence of IBD.^40^ Cariogenic bacteria can also participate in intestinal inflammation. *Streptococcus mutans* TW295 is a serologically κ type (distributed in less than 5% of the population). The bacteria can express the collagen-binding protein (CBP), which is more common in patients with bacteremia after tooth extraction and infective endocarditis.^41^ Animal experiments have found that jugular vein injection of *Streptococcus mutans* TW295 can aggravate the degree of inflammation in a mouse model of colitis.^42^ According to clinical research, the detection rate of this strain in patients with colitis is also higher than that of healthy people.

Oral microbiome studies have found that the oral flora of IBD patients is significantly different from that of healthy people.^43,44^ The number of oral dominant bacteria in patients with IBD has changed, including *Streptococcus*, *Prevotella*, *Neisseria*, *Haemophilus*, and *Veillonella*.^45^ Oral bacteria, such as Campylobacter and *F. nucleatum*,^46^ were detected in tissue samples from IBD patients. The intestinal invasive Campylobacter was detected in 50% of patients with inflammatory bowel disease, but it was not detected in healthy people.^47^ Moreover, the *F. nucleatum* isolated from the inflammatory intestinal epithelium of patients with IBD is more invasive than the strain isolated from healthy people or healthy intestinal epithelium of patients with IBD.^48–50^ In addition, *Klebsiella pneumoniae* from the oral cavity can enter the intestinal tract ectopically, causing an increase in Th1 cells in the intestinal mucosa, thereby inducing IBD.^51,52^ Clinical studies have also found that more oral-derived microorganisms can be detected in the intestinal mucosa and feces of patients with IBD.^42,53,54^ Based on these results, we have deepened our view that the microorganisms in the oral cavity can be ectopic to the intestine to trigger intestinal inflammation.

### Oral microbes and liver diseases

There is a significant difference between the tongue flora of patients with liver cancer and healthy people.^55^ The oral flora of patients with liver cancer is more diverse, and the composition of the flora is significantly different from that of healthy people, and Bacillus, Leptotrichia, Actinomyces, and Campylobacter have higher abundances, while Haemophilus, Streptococcus, and Pseudomonas have lower abundances.^56^ Many clinical studies have demonstrated that periodontitis is a risk factor for non-alcoholic fatty liver, and *P. gingivalis* plays a significant role in the course of non-alcoholic fatty liver.^57–59^ Lanjuan et al. analyzed the intestinal microbiome of patients with liver cirrhosis and found a large number of oral microorganisms, including Veillonella, Streptococcus, Prevotella, Haemophilus, Lactobacillus, and Clostridium.^60^ Therefore, it can also be considered that the invasion of oral microorganisms has been found in the intestines of patients with liver cirrhosis. The invasion of *P. gingivalis* into the intestine can change the composition of the intestinal microbiome,^37^ increase the permeability of the intestinal mucosa and insulin resistance,^61^ lead to the spread of intestinal bacteria to the liver, and increase the content of triglycerides in liver tissue.^58^

## Oral bacteria and cancers

A large amount of research evidence shows that oral microbes play a great role in tumor proliferation, invasion, and metastasis.^3,50,62–70^ Oral microorganisms can directly act on the occurrence and development of tumors through some cytokines and pathways or promote the formation, deterioration, and metastasis of tumors by regulating the immune response between tumors and the body.^71–73^ The oral microbes that play a role in this can be used as potential biomarkers for the study of oral cancers and used to detect the development of oral cancer.^74,75^ An in-depth understanding of the interaction mechanism between oral microbes and tumors will facilitate the design of subsequent new targeted drugs. This will play a great role in the diagnosis, treatment, and prognosis of patients with oral cancer in the future.

### Oral microbes and oral squamous cell carcinoma

Oral cancer and oropharyngeal cancer rank sixth among systemic malignant tumors.^76^ Early detection and treatment are the best strategies to prevent and control oral cancer. Common risk factors for oral tumors include smoking, alcohol abuse, HPV, ultraviolet rays, etc.^77,78^ In recent years, studies have found much important evidence that oral microbes are closely related to OSCC, including *P. gingivalis*,^79^ *F. nucleatum*,^80^ *Treponema denticola*^81,82^ and *Candida*,^83^ etc.

Analysis of *P. gingivalis* in cancerous tissues of OSCC patients and gingival tissues of healthy people found that the content of *P. gingivalis* in OSCC patients was remarkably higher.^84–86^ The staining density of *P. gingivalis* in cancerous tissues was more than 2 times of *Streptococcus gordonii*, and the content of *P. gingivalis* in the tissues is positively correlated with the metastasis of malignant tumors.^87,88^ Microbiome sequencing was used to evaluate the bacterial communities associated with OSCC, and it was found that the diversity and relative abundance of bacteria in the saliva of OSCC patients changed significantly.^74,89^ When used as a diagnostic marker, oral bacteria were found to have 80% sensitivity and 83% specificity between the case group and the control group.^74^

There are many ways in the antiapoptotic mechanism of *P. gingivalis*.^90,91^ It can activate serine/threonine protein kinase (PKB) to phosphorylate the pro-apoptotic protein Bad to form an inactive state. Due to the decrease in the content of the pro-apoptotic protein Bad, the ratio of the antiapoptotic factor B lymphocyte/leukemia-2 protein (Bax) on the mitochondrial surface increases, thereby inhibiting the release of cytochrome oxidase C to inhibiting cell apoptosis.^92^ *P. gingivalis* inhibits activation of caspase-3 by activating Akt and Jak/Stat dual signaling pathways, blocking the increase of mitochondrial membrane permeability caused by caspase-3, and triggering inherent mitochondrial apoptosis.^93,94^ At the same time, *P. gingivalis* can also activate the Jak/Akt/Stat3 signaling pathway to upregulate the anti-apoptotic protein Bcl-2, the tumor formation-related gene c-Myc, and the apoptosis-inhibiting gene Survivin, inhibiting the inherent apoptosis of mitochondria and cell apoptosis.^95^ *P. gingivalis* can also activate the classical signaling pathway-phosphatidylinositol 3-hydroxy kinase (PI3K)/Akt signaling pathway, and activate the expression of NF-κB, participate in a series of processes such as anti-apoptosis, promote angiogenesis and tumor cell invasion, and can further promote anti-apoptosis factor expression.^96^ In addition, *P. gingivalis* up-regulates the expression level of miRNA-203, inhibiting the negative regulatory molecule-cytokine signal transfer to suppressor of cytokine signaling-3 (SOCS3) function, thereby inhibiting cell apoptosis.^96^ *P. gingivalis* invades into the cell and secrets adenosine triphosphate (ATP) enzyme-like nucleoside diphosphate kinase (NDK) to act on extracellular ATP and block its binding to purinergic receptor-7 to inhibit ATP-dependent cell apoptosis and change the normal cycle of cells to make them cancerous.^97,98^

Another mechanism of *P. gingivalis* to promote tumors is to change the local inflammatory microenvironment. The LPS of *P. gingivalis* binds to TLR-2 and TLR-4 on the host cell membrane and then activates NF-κB through the intracellular signal transduction pathway to induce the production of IL-1α, IL-6, TNF-α, and IL-8 promotes the inflammatory microenvironment for tumor growth.^85^ And when NF-κB is improperly activated in the nucleus and cannot return to the cytoplasm, its function can be abnormally increased to change the normal signal transduction of cells and promote cell cancelation.^99^ The up-regulated vascular endothelial growth factor (VEGF) and cyclooxygenase-2 (COX-2) can induce tumor angiogenesis and promote the synthesis of uPA (Urokinase plasminogen activator), involved in the invasion and metastasis of OSCC.^100^ B7-H1 can increase the production of regulatory T cells, which can inhibit killer T cells and help tumor cells to achieve immune escape and promote lymph node metastasis of tumor cells.^101^ *P. gingivalis* induces the expression of B7-H1 and B7-DC receptors on the surface of gingival epithelial cells and squamous cell carcinoma cells.^102^ And also, *P. gingivalis* can interact with PAR-2 and PAR-4 to promote their gene expression.^97^ PAR-2 can activate NF-κB and PAR-4 can promote the phosphorylation of ERK1/2, p38, NF-κB, and thus promote the increase of inactive proMMP9 expression.^103^ *P. gingivalis* further secretes gingipains to promote the activation of proMMP9 and form active MMP9, which participates in the destruction of the surrounding matrix of the cell, promotes the inflammation and the invasion and metastasis of OSCC cells.^104^ Gingipains can exert cytotoxic effects, stimulate host endothelial cells to secrete IL-8, promote tumor angiogenesis and tumor growth, and change T cell functions by degrading T cell surface receptors and enhance OSCC immune escape.^105^

Co-infection of *P. gingivalis* and *F. nucleatum* has a stronger promoting effect on OSCC. Binder et al. established a mouse model with periodontal disease and OSCC and proved that *P. gingivalis* and *F. nucleatum* can directly interact with oral epithelial cells to stimulate tumorigenesis through Toll-like receptors. *P. gingivalis* and *F. nucleatum* trigger TLR signals, leading to IL-6 production, activating STAT3, and STAT3 induces important effect factors to drive the growth and invasiveness of OSCC.^106^ In addition, similar to *P. gingivalis*, infection of *F. nucleatum* alone has also been shown to promote OSCC. In order to study the mechanism of *Fusobacterium* promoting OSCC, Amani et al. used bacteria treated in different ways to infect oral cancer cells. It was found that in addition to increasing the secretion of IL-8 and MMP, oral cancer cells infected with live *F. nucleatum* significantly induced higher expression of STAT3, MYC, and ZEB1.^107^ This indicates that in addition to promoting tumor proliferation through Toll-like receptors, *F. nucleatum* can also promote tumor invasion and epithelial–mesenchymal transition.

*T. denticola* is also known to be associated with the development and progression of oral cancer.^108^ In vitro studies have found that Td-CTLP, a virulence factor of *T. denticola*, is detected in tongue squamous cell carcinoma and may regulate tumor-associated immunomodulatory proteins, such as upregulation of MMP-8 and MMP-9, promote the occurrence and development of tumors.^109^ Listyarifah et al. also found that Td-CTLP is significantly present in early tongue squamous cell carcinoma (MTSCC), and its expression is related to tumor invasion depth, tumor diameter, TLR-7, TLR-9 expression. This may suggest that Td-CTLP can be used as a marker for *T. denticola* to promote OSCC.^110^ Based on the above studies, the authors have studied the mechanism of *T. denticola* promoting tumors. It was found that *T. denticola* regulates the cell cycle by activating the TGF-β pathway, inhibiting cell apoptosis, and promoting the proliferation of oral squamous cell carcinoma.

### Oral microbes and esophageal cancer

Esophageal cancer is one of the most common cancers in the world. In China, the mortality rate of esophageal cancer is as high as 7.75 per 100,000 persons.^111,112^ Poor oral hygiene is considered a risk factor for esophageal squamous cell carcinoma and esophageal squamous epithelial dysplasia. Esophageal squamous cell carcinoma is closely related to oral conditions such as tooth loss and tooth brushing frequency. The incidence of metastasis of esophageal squamous cell carcinoma in periodontitis patients is significantly higher than that in non-periodontal patients.^113^ However, the specific mechanism of this correlation is not yet clear.

The microecological imbalance caused by poor oral hygiene may be one of the reasons that cause the accumulation of carcinogens, and the body is in a state of continuous inflammation. Research by Narikiyo et al. found that periodontal disease-causing bacteria *T. denticola* can be detected in tumors and normal tissues of patients with esophageal cancer, and further promote tumor formation through inflammation.^82,114^ Chen et al. sequenced the microbial 16s rRNA gene of saliva samples from esophageal squamous cell carcinoma and healthy people and found that the overall species diversity of salivary microorganisms in patients with esophageal squamous cell carcinoma decreased, the abundance of Corynebacterium, Peptococcus, and Cardiobacterium was also significantly reduced.^115^ The results of these studies show that salivary microorganisms can reflect the changes in the microbial community of patients with esophageal squamous cell carcinoma and that salivary microorganisms may assist in the diagnosis of esophageal cancer.

Further research found that in the lesion area of esophageal squamous cell carcinoma, the detection rate of *P. gingivalis* was higher, 61%, only 12% in adjacent parts, and no normal parts were detected.^116^ At the same time, it was found that the detection of the lysine-specific gingival protease secreted by *P. gingivalis* at the above sites also has similar characteristics. Most patients with severe esophageal cancer are positive for *P. gingivalis*.^117^ Therefore, the colonization of *P. gingivalis* in the esophagus is not only related to the occurrence of esophageal squamous cell carcinoma but also closely related to the severity of squamous cell carcinoma. At the same time, Neisseria is also believed to affect the occurrence of upper respiratory and digestive tract cancers due to its strong activity in metabolizing alcohol to acetaldehyde.

### Oral microbes and pancreatic cancer

A retrospective study compared the prevalence of pancreatic cancer in 139,805 patients with periodontal disease and 75,085 patients without periodontal disease and reached similar conclusions.^118^ Fan et al. compared the oral microbiota of 361 pancreatic cancer patients and 371 non-pancreatic cancer patients and found that the detection rate of *P. gingivalis* and *Aggregatibacter actinomycetemcomitans* was higher in the pancreatic cancer patients.^69^ Another study used high-throughput sequencing technology to analyze the composition of the salivary flora of 108 individuals (8 patients with pancreatic cancer, 78 patients with other diseases, and 22 healthy people).^119^ It also found that the abundance of *P. gingivalis* is relatively high in the saliva of patients with pancreatic cancer. Salivary bacterial DNA detection studies have found that Bacteroides and Granulicatella are more common in patients with pancreatic cancer, but Neisseria, Streptococcus, Corynebacterium, and Bacillus are less numerous.^120^ In patients with pancreatic cancer, more Leptotrichia and fewer Porphyromonas can be detected, which can be converted into a ratio with marker significance (Leptotrichia: Porphyromonas/L:P), a high ratio of L:P has a potential diagnostic value for pancreatic cancer.

### Oral microbes and colorectal cancer

Colorectal cancer is one of the most malignant tumors in the world.^121^ Numerous clinical studies have found that Clostridium and *F. nucleatum* is enriched in colorectal cancer, suggesting that *F. nucleatum* is closely related to the occurrence of colorectal cancer.^50,122–124^ It was confirmed that *F. nucleatum* can bind to E-cadherin on the surface of colorectal cancer cells through the Adhesin FadA on its surface, thereby invading cancer cells and promoting the proliferation of cancer cells.^125^ Fap2 expressed by *F. nucleatum* interacts with TIGHT on the surface of human immune cells to inhibit the activity of immune cells such as T cells and natural killer cells, thereby inhibiting the killing of colorectal cancer cells by immune cells.^126^ It can also affect cell autophagy and promote the development of tumors. Animal experiments have found that continuous oral vaccination or tail vein injection can colonize and accumulate *F. nucleatum* in colorectal cancer, thereby promoting the occurrence and development of colorectal cancer.^120^

## Oral microbes and cardiovascular diseases

Cardiovascular disease refers to diseases that occur in the heart and vascular circulatory system, including coronary heart disease, endocarditis, and myocardial infarction.^127^ Atherosclerosis (AS) is an important pathological process of coronary heart disease. It is closely related to the proliferation of vascular smooth muscle cells and the functional changes of vascular intimal cells. The main pathological manifestation of AS is lipid deposition in the vascular endothelium of the large and middle arteries, forming scattered or flakes of atherosclerotic plaques, resulting in narrowing of the arterial lumen.^128^ Many cross-sectional studies, case analyses, and epidemiological investigations have found that periodontitis is an important risk factor for cardiovascular disease.^129–131^ The gingival epithelium in the periodontal pockets of patients with periodontitis is prone to breakage, which helps bacteria to enter the systemic circulatory system, leading to bacteremia, or ectopic colonization in other organs in the body. Periodontal disease-related bacteria can destroy the body’s immunity, stimulate cells to produce inflammatory factors such as IL-1β, IL-6, TNF-α, and enter the blood circulation from the damaged periodontal tissue, resulting in inflammation and vascular endothelial damage, and the formation of atherosclerotic plaques.^132,133^ Animal experiments have found that *P. gingivalis* inoculated into the mouth of mice can cause an increase in the number of Bacteroides and a decrease of Firmicutes, which are closely related to endotoxemia and systemic inflammatory reactions.^134^ Many clinical studies have found that after the periodontitis of the patient is controlled, the development of AS can be suppressed accordingly.^135^ It has been reported that the increase in the level of C-reactive protein in the body caused by oral bacterial infection has an important correlation with the development of atherosclerotic vascular disease.^136^ Lactobacillus and Streptococcus in the oral cavity are related to the occurrence of infective endocarditis. *Streptococcus sanguis* is an early colonizer of dental plaque, with the highest detection rate in the endocardium of patients, and is closely related to the inflammatory process of endocarditis.^137^ The pathogenesis may be that the thrombus-like protrusions formed on the surface of the vascular endothelium promote the adhesion of bacteria, which in turn leads to the occurrence of infection. In the animal model study of endocarditis, the virulence factor of *S. gordonii* is significantly increased, indicating that the virulence factor of oral bacteria may relate to the process of endocardial infection.^138^

After the treatment of periodontitis, C-reactive protein, which is an indicator of the improvement of the systemic inflammatory state, was significantly reduced in the patient’s serum, and some other serum inflammatory factors were also significantly reduced.^139,140^ Therefore, the state of periodontal inflammation is directly related to the inflammatory factors in the serum. The gingival epithelium in the periodontal pockets of patients with periodontitis is prone to breakage, which helps bacteria to enter the systemic circulatory system, leading to bacteremia, or ectopic colonization in other organs in the body. *P. gingivalis* can invade and colonize the patient’s atherosclerotic plaque.^141^ Chiu et al. analyzed the microorganisms in carotid endarterectomy specimens of 76 cardiovascular patients and found that the positive detection rate of *P. gingivalis* was 42%.^142^ And it was also detected other periodontal pathogens in atherosclerotic plaques, such as *A. actinomycetemcomitans* and *T. forsythia*.^143^ Periodontal disease-related bacteria can stimulate cells to produce inflammatory factors such as IL-1β, IL-6, TNF-α, and enter the blood circulation from the damaged periodontal tissue, causing related inflammation and promoting the formation of atherosclerotic plaques.^144^ Animal experiments found that after infection of *P. gingivalis* in ApoE gene-deficient mouse models, the onset of AS near the aorta and the aortic trunk was earlier and the damage was more serious.^145^ *P. gingivalis* inoculated into the mouth of mice can cause an increase in the number of Bacteroides and a decrease in the number of Firmicutes, which can cause severe immune inflammation in a short period, leading to a sharp increase in the level of serum inflammatory factors.^146^ Many clinical studies have found that after the periodontitis of the patient is controlled, the development of AS can be suppressed accordingly.^147,148^ Since Galectin-3 is expressed on the surface of cells and is readily secreted by damaged and inflammatory cells, it is considered to be a biomarker of heart inflammation and fibrosis. Gaetano et al. analyzed the association and influence of periodontitis and coronary heart disease (CHD) on saliva and serum Galectin-3 and suPAR, a soluble urokinase-type plasminogen activator receptor, in patients with periodontitis and CHD. The results showed that patients with periodontitis and periodontitis + CHD showed significant serum Galectin-3 and suPAR levels.^149,150^ Therefore, periodontitis is an important predictor of serum Galectin-3 and suPAR levels associated with CHD. Based on the above-mentioned large number of research results, the American Periodontology Association and the European Periodontology Association agree that periodontal inflammation is a risk factor for cardiovascular disease, and periodontal pathogens or bacterial metabolites entering the blood circulation may directly induce AS.^136^

## Oral microbes and Alzheimer’s disease

The relationship between oral microbes and Alzheimer’s disease has attracted people’s long-term attention. Clinical investigations and studies have provided some evidence for the causal relationship between periodontitis and Alzheimer’s disease. In a longitudinal cohort study, 152 subjects in the age range of 50–70 years were followed up for 20 years. The results found that the severity of periodontitis and the cognition level is in an inverse relationship in subjects with fewer than 10 teeth missing.^151^ Another longitudinal aging cohort study of 144 subjects showed that people with the APOE-ε4 gene and fewer teeth have a faster cognitive decline than people without these two risks.^152^ In addition, a longitudinal cohort study conducted a 32-year survey of 597 male individuals. The results showed that tooth loss, the depth of periodontal pockets, and the degree of alveolar bone loss are related to cognitive impairment, especially when they are older than 45 years old.^153^ A prospective follow-up survey of 5468 subjects found that irregular tooth brushing habits are also related to Alzheimer’s disease.^154^ A survey in the United States found that there is an association between periodontitis and cognitive impairment in 2355 60-years and older people.^155^ After adjusting for the level of education and other confounding factors, Poole et al. found that among 5138 subjects aged between 20 and 59, gum bleeding and periodontal attachment loss have a strong correlation with cognitive impairment.^151^ Because chronic periodontitis and Alzheimer’s disease are both multifactorial diseases, they are affected by a variety of factors, and they have many common risk factors, such as smoking and education level. Therefore, the causal relationship between tooth loss or periodontal disease and cognitive impairment has not been fully confirmed.

Studies found that periodontitis induced by *P. gingivalis* increased the deposition of Aβ in the brain, and the levels of IL-1β and TNF-α, which affected the cognitive ability of mice.^156^ Another study induced experimental chronic periodontitis by orally feeding 10 C57BL/6 mice with *P. gingivalis* or gingipains for 22 weeks. It was found that mice showed neurodegenerative diseases similar to Alzheimer’s disease and formed extracellular Aβ1-42.^157^ Stephen et al. found that *P. gingivalis* can colonize the brain of mice and increase the accumulation of Aβ1-42.^158^ It was also found that gingipains, the virulence component of *P. gingivalis*, is neurotoxic in vivo and in vitro, and harms Tau protein (a protein required for normal neuronal function). Inhibiting gingipains by using small molecule inhibitors can reduce the bacterial burden of *P. gingivalis* in brain tissue, prevent the production of Aβ1–42, reduce neuroinflammation, and reduce hippocampal neuronal damage.

We speculate that oral microbes can affect brain tissue in two ways. One is that pro-inflammatory cytokines caused by periodontal bacteria enter the brain tissue through the systemic circulation. The permeability of the blood-brain barrier is believed to increase with age and predates Alzheimer’s disease. This has been confirmed in animal models. The amyloid precursor protein gene mutations in genetically modified mice (5XFAD mice) have increased blood-brain barrier permeability and increased senile plaque accumulation.^159^ As the mice age with growth, this effect becomes more obvious.

The other is that periodontal microorganisms or their products enter the central nervous system through peripheral nerves, such as the glossopharyngeal and/or trigeminal nerve.^160^ The detection of oral spirochetes in the trigeminal ganglion also supports this. In addition, the ventricular tissues are not protected by the blood–brain barrier, so they may also become the entrance for bacteria to enter the brain.

There is a two-way relationship between chronic periodontitis and Alzheimer’s disease. Due to limited mobility and poor oral hygiene in patients with Alzheimer’s disease, it promotes the accumulation of periodontal tissue inflammation and eventually leads to tooth loss. Because the loss of teeth affects the patient’s eating and nutritional status, it may worsen the patient’s memory and other nervous system functions. These have been confirmed in animal experiments. Therefore, the relationship and mechanism of the interaction between periodontitis and Alzheimer’s disease need to be further studied.

## Oral microbes and diabetes

Diabetes is a common endocrine and metabolic disease caused by insulin deficiency, impaired pancreatic islet function, or impaired insulin biological action.^161^ Diabetes with poor blood sugar control is a risk factor for periodontal diseases, and periodontitis is the sixth major complication of diabetes.^162^ Compared with non-diabetic patients, the risk of chronic periodontitis in diabetic patients is increased by 2–3 times.^163^ The pathological changes of diabetes can aggravate and accelerate the occurrence and development of periodontal inflammation, and the effective control of periodontitis has a pivotal effect on the control of blood sugar. The oral microbes in the periodontal pockets of patients with periodontitis have complex interactions with the human immune system, resulting in continuous chronic inflammation. Compared with non-diabetic periodontitis patients, the community structure of the subgingival microbiome of diabetic and periodontitis patients has undergone significant changes, and a variety of bacteria between the two are differentially enriched.^164^ Oral microorganisms may trigger insulin resistance by influencing the body’s immune inflammation and oxidative stress, thereby affecting the process of diabetes.

Oral microbes can affect the occurrence and development of diabetes by regulating systemic immune homeostasis.^165^ Microbial infection can stimulate the immune response of local periodontal tissues and produce large amounts of inflammatory cytokines. For example, the excessive response of mononuclear macrophages to periodontal bacteria can lead to the local production of high concentrations of IL-1 β, IL-6, and TNF-α in periodontitis.^140,166^ These cytokines can enter the circulatory system and affect multiple system tissues and blood vessels throughout the body. The endotoxin of *P. gingivalis* binds to the CD14 molecules on the surface of macrophages to activate the TLR2/4 signaling pathway.^167^ Activated TLR2/4 can bind to the TIR homology domain and the N-terminal death domain of MyD88 to activate IRAK. The activated IRAK then binds to TNF receptor-related factors to activate JNK, MAPK, and NF-κB signal transduction pathway eventually leading to a chronic inflammatory state. NF-κB can inhibit the expression of some important proteins in the insulin signaling pathway, such as glucose transporter 4 protein (GLUT4).^168^ TNFs stimulate fat cells to break down lipids, thereby increasing the level of free fatty acids in the blood and reducing insulin sensitivity. Persistent inflammation will lead to insulin resistance, which can further aggravate the systemic inflammatory response and cause a long-term imbalance of the inflammatory axis, affecting blood glucose metabolism. In addition, Bhat et al. found that endotoxin stimulation by *P. gingivalis* up-regulates the expression of immune-inflammatory response-related genes (Cd8a, Cd14, and Icam1) and insulin signaling pathway-related genes (G6pc and Insl3) of the pancreatic β-cell line MIN6.^169^ Animal experiments have also confirmed that the dysbacteriosis caused by periodontitis can promote the occurrence of insulin resistance in mice on a high-fat diet through an adaptive immune response.

Oxidative stress is an important way for host immune defense and microbial killing. However, in the absence of a compensatory endogenous antioxidant response, a large amount of ROS produced by oxidative stress can cause body damage and affect the occurrence and development of diabetes.^170^ Gram-negative bacteria and their metabolites in the subgingival microenvironment of patients with periodontitis can enter the periodontal tissue through the broken and loose gingival sulcus epithelium and spread to the surroundings, leading to the production of large amounts of ROS in inflammatory cells and vascular endothelial cells. ROS can interfere with the insulin signal transduction pathway, causing insulin resistance. Insulin resistance will increase the level of ROS, which will further damage the metabolic and vascular factors in the insulin signal transduction pathway. Oxidative stress interferes with the insulin signaling pathway by inhibiting tyrosine phosphorylation and activates a variety of serine/threonine kinase cascades through NF-κB activating enzymes, p38MAPK, JNK/SAPK, and protein kinase C (PKC) isoenzymes.^171^ These cascade reactions phosphorylate the free serine/threonine sites of related enzymes in the insulin signaling pathway, thereby disrupting the insulin signaling pathway.

In the state of periodontitis, periodontal pathogens enter the blood circulation directly or indirectly, which can cause bacteremia, and may colonize the distal end of the body, thereby inducing systemic inflammation. The systemic inflammatory state caused by periodontitis is different from the direct invasion of bacteria. The oral inflammatory and periodontitis-related pathogens are more likely to be the initiators of the systemic inflammatory. Patients with periodontitis have elevated levels of systemic inflammatory mediators, such as c-reactive protein, pentaxin-3, and fibrinogen.^172^ Under active periodontal treatment intervention, these systemic inflammation biomarkers can be significantly down-regulated.^173^ Therefore, the disordered oral flora of patients with periodontitis can not only mediate the periodontal inflammatory state but also affect the systemic inflammatory state of the distal body.

## Oral microbes and rheumatoid arthritis

Rheumatoid Arthritis is an autoimmune inflammatory disease with chronic, symmetric, polysynovial arthritis and extra-articular lesions as the main clinical manifestations.^174^ Patients with rheumatoid arthritis have a higher incidence of periodontitis and are usually accompanied by more serious periodontal inflammation. Effective treatment of periodontal disease has a positive effect on the control of rheumatoid arthritis. It is worth noting that rheumatoid arthritis and periodontitis have similar risk factors, such as human leukocyte antigen HLA-DRB1 allele polymorphism and smoking.^175^ Both have similar pathological manifestations, such as chronic inflammation and bone resorption mediated by IL-1, TNF-α, and matrix metalloproteinases. Therefore, we believe that rheumatoid arthritis and periodontitis may have similar immune-inflammatory causes. Since periodontitis is an immune-inflammatory response induced by oral microbial infection, it is speculated that oral microbial infection may be involved in the pathological process of rheumatoid arthritis as an environmental factor. In recent years, with the continuous deepening of research, the related mechanism of the relationship between oral microbes and rheumatoid arthritis has also been continuously gained new understanding.

Anticitrullinated protein antibodies (ACPA) are highly specific immunoglobulins in patients with rheumatoid arthritis.^176^ This antibody is considered to be an important factor linking rheumatoid arthritis and periodontitis. Citrullination is a post-translational repair mechanism in the body. It is catalyzed by peptidyl arginine deiminase (PAD), which can change the biochemical properties and immunity of the host protein, resulting in the generation of new antigenic determinants and inducing the body to produce autoantibodies against these epitopes. *P. gingivalis* gingipains and peptidyl arginine deiminase (pPAD) can lead to citrullination of the host protein, which in turn induces autoantibody reactions.^177^ Gingipain is a cysteine-like protease that can catalyze the cleavage of arginine at the C-terminus of polypeptides, and the resulting free arginine can be further citrullinated by pPAD. pPAD has a unique domain, does not have homology with mammalian PAD, and has different functions from mammalian PAD.^178^ Therefore, the citrullination catalyzed by pPAD may generate new epitopes and antigenic determinants. Although pPAD homologs can be detected in a variety of microorganisms in the oral cavity, intestines, and environment, *P. gingivalis* is the only bacteria that have been found to carry functional PAD at present. In addition, after collagen-induced arthritis mice were infected with *P. gingivalis*, the production of anti-collagen type II antibodies and the increase in inflammation of arthritis in mice could be detected.^179^ This kind of phenomenon does not appear in the same animal model of infection with the pPAD gene knocked out strain of *P. gingivalis*. Significantly, because pPAD can be automatically citrullinated, specific IgG antibodies against citrullinated pPAD can be found in patients with rheumatoid arthritis. And this antibody cannot be detected in healthy people or patients with chronic periodontitis. This suggests that pPAD itself may be one of the antigenic substances that cause RA immune inflammation. However, the titer of anti-pPAD antibodies in humans is extremely low and hard to be detected. However, the biomarkers of cascade amplification induced by pPAD may become very potential clinical markers for RA.

There is also a certain correlation between *A. actinomycetemcomitans* induces the imbalance of the PAD activity of neutrophils by secreting LtxA, which leads to high citrullination of proteins in neutrophils and the formation of self-antigens.^179^ Ltx A can also change the morphology of neutrophils, mimic the formation of neutrophil extracellular traps and cause neutrophil lysis, and finally release a large amount of citrullinated antigen. Different from *P. gingivalis* self-synthesis of bacterial PAD, *A. actinomycetemcomitans* mainly induces endogenous PAD activity, leading to the formation of autoantigens, and ultimately the formation of ACPA and rheumatoid factor and the occurrence of RA.

The oral microbial composition and community function of patients with rheumatoid arthritis are different from those of healthy people, and these differences are related to the clinical index. The researchers used 6 plaque MLG and 2 salivary flora information to fit the diagnosis model, and the specificity and accuracy of disease diagnosis were higher than 80%.^180^ The diagnostic model constructed by integrating community information of the intestine, plaque, and saliva has better accuracy and specificity. This indicates that the oral microbial composition of patients with rheumatoid arthritis is finely regulated by the disease. The oral microbiome affects the development and prognosis of RA and has extremely high sensitivity, which can be used as a fingerprint for disease progression and prognosis assessment.

## Oral microbes and preterm birth

Preterm birth is the delivery of a fetus under 37 full weeks of pregnancy or 259 days of gestation.^181^ In China, the birth of a fetus at 28 full weeks of gestation or with a newborn weight of ≥1000 g but less than 37 weeks is regarded as preterm delivery.^182^ Newborns born during this period with a weight of 1000–2499 g and immature body organs are considered preterm babies.^182^ Approximately, 35% of neonatal deaths worldwide are related to preterm birth.^183^ Even if preterm babies survive, there may be complications such as mental retardation, abnormal vision, and hearing, which seriously increases the burden on the family and society. However, the reasons for preterm birth are unknown and there are many inducements, making it difficult for its prevention and treatment. Some oral common microorganisms were detected in the placenta, which indicates that the mechanism of preterm delivery can be explored from the oral conditions of pregnant women.^184^

The results of metagenomic sequencing of the placental microbial samples showed that the composition of the placental microbiome is most similar to the oral microbiome when compared with the vagina, intestine, and respiratory tract.^184^ Based on this, it is speculated that the bacteria in the oral microecology may colonize the placenta, and its virulence factors may produce pathological effects in the local placenta and induce preterm delivery. Researchers injected saliva and subgingival plaque obtained from the population into the tail vein of the mice, and then tested the bacterial composition in the placenta, and found that the bacteria colonized in the placenta are mostly oral symbiotic bacteria, and their colonization has a certain specificity, rather than physical diffusion.^184^ The difference in the composition of the oral microbiota at the placental site between preterm delivery and full-term delivery may lead to adverse pregnancy outcomes such as preterm delivery.

To study the effects of *P. gingivalis* on adverse pregnancy outcomes, Collins et al. injected the lipopolysaccharide of *P. gingivalis* into golden hamsters and found that both pre-pregnancy injections and injections during pregnancy had harmful effects on fetal mice with a dose-dependent effect.^185^ They also used the Chamber research model to repeat the above experiment and found a rising rate of adverse pregnancy outcomes and an increase of PGE2 and TNF-α in the LPS treatment mice.^186^ This indicates that *P. gingivalis* may induce premature delivery in mice by triggering acute and chronic inflammatory reactions. Lin et al. found that *P. gingivalis* can be detected in the liver, uterus, and placenta of the mothers of growth-restricted mice with increasing serum TNF-α, IFN-γ and *P. gingivalis* specific antibodies IgG.^187^ This indicates that *P. gingivalis* can stimulate the mother to produce an inflammatory response, leading to intrauterine growth restriction.

It has also been reported that in a case of a pregnant woman with gingivitis and upper respiratory tract infection who gave birth to a stillbirth, the 16S rRNA coding sequence analysis method was used to detect *F. nucleatum* from the placenta and the fetus, and its sequence was similar to that of the maternal gingival *F. nucleatum*.^188^ This case shows for the first time that *F. nucleatum* may be transferred from the human oral cavity to the placenta. In the experiment of injecting *F. nucleatum* into the veins of mice, it was also observed that *F. nucleatum* infects the mouse placenta in the form of biofilm and causes premature delivery and even stillbirth.^189^ Experiments using *F. nucleatum* and TLR-4 or TLR-2 deficient mice found that TLR-4 deficient mice had lower fetal mortality and less placental inflammation, while TLR-2 deficient mice did not. At the same time, the treatment of wild-type mice with TLR-4 antagonists can reduce fetal mortality and reduce the occurrence of decidual necrosis without affecting the colonization of placental bacteria. This indicates that the inflammation caused by oral microorganisms may be more important than the role of bacteria itself for adverse pregnancy outcomes such as preterm birth.

In addition, Bergerella was also detected in the amniotic fluid analysis of the parturient, and its 16S rRNA coding sequence was consistent with that in the subgingival plaque of the parturient but was not detected in the vagina.^190^ This finding confirmed that the bacteria in the oral cavity entered the amniotic fluid and suggested that Bergerella may be related to preterm birth. Other oral bacteria detected in the placenta and amniotic fluid include *Capnocytophaga gingivalis, Listeria monocytogenes, Tannerella forsythia, T. denticola, Peptostreptococcus micros, S. sanguis*, and *A. actinomycetemcomitans*. Whether these bacteria have the effect of causing preterm birth and the specific mechanism remains to be further studied. The proliferation of these oral microorganisms at the placenta may break the ecological balance of local sites cause pathological alterations and induce preterm birth. However, the abnormal proliferation of these oral microorganisms at the placenta may induce the host’s inflammatory response. Compared with the bacteria themselves, the release of inflammatory cytokines may play a more important role in causing adverse pregnancy outcomes.

## Conclusions and perspectives

The relationship between the structure and function of oral microbes and the balance of human health and disease is becoming clearer. The rapid development of high-throughput sequencing technology and bioinformatics technology has made it possible to comprehensively study the composition of oral microbes. Since the human body is a “super complex” composed of human cells and microorganisms, microorganisms, as the second genome that affects human health, can settle inside and on the human body. As an important part of the human microbiome, the oral microbiome is systemic and community-specific. However, compared with the gut microbiome, the study of the oral microbiome is still in its infancy. Many studies have focused on the discovery of microbial diversity, and few have dealt with the impact of community function, host genetic background, lifestyle, and biological function events on the oral microbiota. The lack of these data has greatly affected people’s comprehensive understanding of the oral microbial community. In addition, under the promotion of HMP and other microbial metagenomics research projects, the use of high-throughput sequencing technology to understand the oral microbial community has continued to deepen, and massive amounts of big data information have been obtained. How to effectively transform biological big data into clinical diagnosis and treatment methods with practical application value, and then provide patients with effective individualized medical services, there are still many problems to be solved urgently. The continuous development of metagenomics technology and high-throughput sequencing technology has greatly expanded human understanding of the relationship between the oral microbiome and systemic diseases. Oral microbiology has changed from studying the pathogenicity of individual bacteria to the relationship between oral microecological balance and systemic diseases. Understanding the specific mechanisms that maintain and regulate oral microecological balance is of great significance to the prevention and treatment of oral diseases and even human systemic diseases.
